# A Systems View of *Ganoderma* Triterpenoids Biosynthesis: Signals, Transcription, and Biosynthetic Remodeling

**DOI:** 10.3390/jof12100795

**Published:** 2026-10-21

**Authors:** Meilin Cui, Xueting Gao, Wei Zhao, Jiali Wang, Yanfang Wu, Xiuhong Zhang

**Affiliations:** College of Food Science, Shanxi Normal University, Shanxi Engineering Research Center of Microbial Application Technologies, Taiyuan 030031, China

**Keywords:** *Ganoderma* triterpenoids, signal transduction, transcription factors, engineering strategies, multi-omics

## Abstract

*Ganoderma* triterpenoids (GTs), the major bioactive metabolites of *Ganoderma lucidum*, are widely known for their pharmaceutical versatility. However, their natural biosynthesis ability is insufficient to meet industrial demands, so it is urgently required to clarify the metabolic network of GTs and develop new strategies to increase the total GTs production. Recently, the complex multi-tiered regulatory framework, which includes the sequential integration of environmental perception, signal transduction, and transcriptional reprogramming, has been fully dissected. This review synthesizes recent breakthroughs that illustrate how environmental stimuli are transduced through independent or intricate spatiotemporal interactions involving reactive oxygen species (ROS), calcium ions (Ca^2+^), and nitric oxide (NO). Such interplays give rise to oscillatory or feedback loops that precisely modulate metabolic production. Moreover, we systematically examine various transcription factors that control metabolic fluxes by either directly targeting the rate-limiting enzymatic steps of the mevalonate (MVA) pathway and downstream CYP450-modification networks, or via epigenetic and complex-mediated pathways. Importantly, we consider the application of gene editing, AI/ML-guided enzyme engineering, and subcellular compartmentalization as innovative ways to overcome their yield bottlenecks. Successful heterologous construction of biosynthetic pathways highlights the continuing trend towards predictability in biomanufacturing. In summary, this review presents a holistic translational approach from foundational regulatory mechanisms to synthetic assembly, establishing *G. lucidum* as a viable and intelligent bio-manufacturer for producing high valuable GTs.

## 1. Introduction

*G. lucidum* is a laccate, shelf-forming polypore white-rot basidiomycete belonging to Basidiomycota, Polyporales and Ganodermataceae. It is an extremely valuable edible and medicinal fungus that has been extensively utilized as an ingredient of traditional Chinese herbal medicine for more than 2000 years. Extensive research has demonstrated its unique pharmacological properties, like antitumor, hepatoprotective, anti-inflammatory, bidirectional immunomodulatory, lipid- and glucose-regulating, and histamine release-inhibiting effects [1,2,3]. In recent times, advancements in the whole-genome sequencing of *G. lucidum* and the establishment of its genetic manipulation systems have made this fungi an excellent candidate to study morphological development and secondary metabolic regulation in higher basidiomycetes [4].

To date, more than 400 compounds have been identified and reported from *G. lucidum*, with highly oxygenated lanostane triterpenoids and polysaccharides representing the two major classes of pharmacologically active compounds. With the diversity of their lanostane backbones and modifications by multiple side chain groups, more than 200 ditinct *Ganoderma* triterpenoids (GTs) have been isolated and characterized [5]. Various physiological of GTs resulting from these structural variations are closely linked to the well-recognized properties of *G. lucidum*, which include anti-tumor, anti-inflammatory, liver protective, and hypoglycemic functions [5,6]. Apart from these biological functions, GTs metabolism also plays an important role in critical physiological processes, such as signal transduction, environmental responses, and cellular structure stabilization [5,7,8,9]. GTs content has emerged as an important criterion for assessing the quality of *G. lucidum* [10]. With the increase in the demand for standardized, highly bioactive *G. lucidum* products for use in the nutritional supplements and pharmaceutical industries, new strategies are urgently required to increase GTs yields.

Over the last decade, research into GTs biosynthesis has undergone a paradigm shifting from the pathway study to the regulatory analysis, and finally to the engineering-based reconstruction process. The early studies mainly concentrated on cloning, heterologous expression, and functional validation of key enzymes-encoding genes in the mevalonate (MVA) pathway, which elucidated the complete enzymatic steps from acetyl-CoA to lanosterol [11,12,13]. However, the low yield of GTs has revealed an important bottleneck: since the regulation of GTs production is very complex and multilevel, the availability of structural genes is insufficient to guarantee high metabolic flux. Therefore, understanding this regulatory network will provide a rational foundation for improving GTs production through genetic or metabolic interventions.

The GTs biosynthesis controlled in various manners involving the interaction of environmental factors (temperature, pH, nutrients, etc.) [4,14,15], intracellular signal molecules (such as reactive oxygen species [ROS], calcium ions [Ca^2+^], and nitric oxide [NO]) [16,17,18], and transcriptional regulatory networks [8,19]. Progress in the field of genomic annotation and functional genomics tools like clustered regularly interspaced short palindromic repeats/CRISPR-associated protein 9 (CRISPR/Cas9) [20], RNA interference (RNAi) [21,22], and DNA affinity purification sequencing (DAP-seq) [23], have greatly enlarged our ability to study the transcriptional regulation of GTs biosynthesis. Recent research on GTs biosynthesis in *G. lucidum* has mainly concentrated on engineering active metabolic pathways, and more and more attention has been paid to developing unnatural GTs biosynthesis pathways. The use of CRISPR/Cas9-based gene editing technology, pathway modularization by means of synthetic biology, and artificial intelligence (AI)- and machine learning (ML)-assisted multi-omics analysis have brought revolutionary change to this field by facilitating precise genetic manipulation, programmable metabolic reconstruction, and data-guided rational optimization [24,25,26,27].

This review provides a summary of the current understanding of GTs biosynthesis in *G. lucidum*. We first introduce how multi-omics approaches enable the systematic dissection of the regulatory networks that govern GTs production. Then, we will focus on the roles of the signal molecules ROS, Ca^2+^, and NO, as well as the transcription factors responsible for translating these signals into metabolic processes. Lastly, we survey the emerging technologies such as CRISPR-based gene editing, synthetic biology, organelle engineering, and ML, which have been used to build GTs biosynthesis as a viable platform for rational metabolic engineering and sustainable production. The purpose of this paper is not only to give a general theoretical framework for both basic research and industrial development of GTs, but also to serve as a practical guide for studying the secondary metabolism in other medicinal fungi.

## 2. Genome of *G. lucidum*

**Figure 1:**
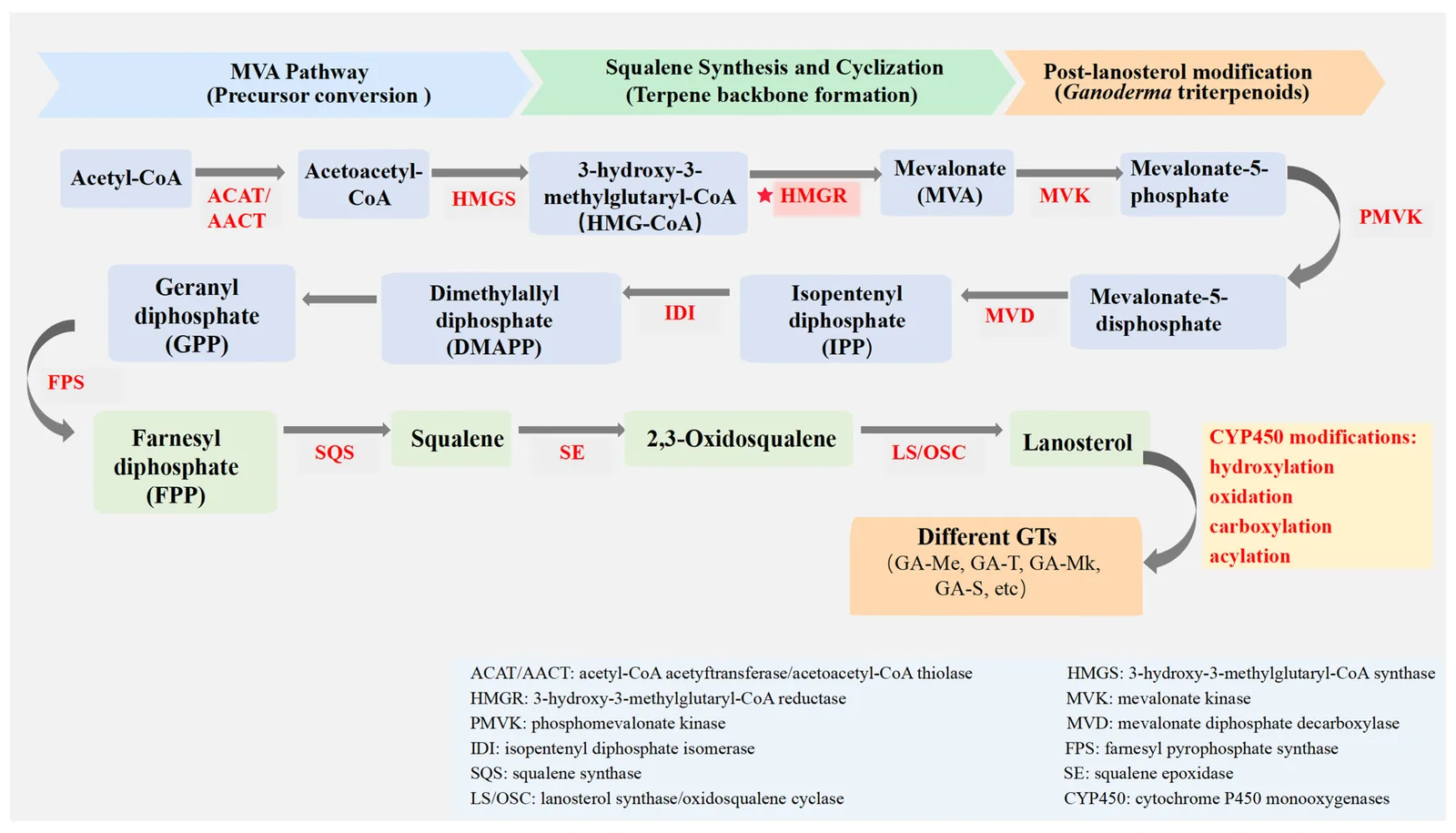
Biosynthetic pathway of GTs via the MVA pathway.

GTs biosynthesis occurs via the classical MVA pathway. Upstream of the cyclic intermediate lanosterol, the structural genes responsible for enzymes’ functions have been successfully cloned and characterized. These genes constitute the core pathway for GTs backbone synthesis [19]. Transcriptomic and proteomic studies have demonstrated that genes such as *AACT*, *HMGS*, *FPS*, and *SQS* exhibited relatively higher expression levels across different developmental stages of *G. lucidum* strains [28]. Among these genes, *HMGR* is an enzyme known to be rate-limiting in MVA pathway. Overexpression of *HMGR* led to a remarkable increase in GTs production, underscoring its essential function in GTs biosynthesis [29]. The increased expression levels of *SQS* and *LS* emphasize their crucial roles in GTs biosynthesis [30,31]. After the lanosterol backbone formation, the post-modifications are mainly catalyzed by CYP450, which enable hydroxylation, oxidation, and other oxidative reactions, causing the GTs diversity (Figure 1) [32].

The first reference genome of *G. lucidum* was derived from the single haploid monokaryon (strain 260125-1), which spans 43.3 Mb on 13 chromosomes, with a GC content of 55.9% and 16,113 predicted protein-coding genes [33]. Other assemblies of *G. lingzhi* range from 49.15 Mb with 13,125 predicted protein-coding genes [34] to 60.56 Mb with 16,592 genes [35]. This spread in genome size reflects strain-level differences in repeat and transposable-element content as well as differences in sequencing platforms and assembly completeness, rather than assembly quality alone. Gene features relevant to secondary metabolism are consistent across these assemblies: a large complement of cytochrome P450 monooxygenases (CYP450). The genome carries a total of 219 CYP450 genes in 42 families, of which, among which 78 upregulated CYP genes are co-expressed with *LS*, while 16 are highly similar to fungal CYPs that hydroxylate steroidal substrates, indicating their involvement in the downstream modifications of lanosterol and, consequently, GTs structural diversity [33,36]. Similarly, the genome of the *G. lucidum* strain Lingjian-2 contains 18 genes that participate in the terpenoid backbone biosynthesis pathway, with the CYP450 gene count reaching 300 [37]. The fraction of the genome devoted to secondary metabolism is mainly distributed across CYP450, terpene cyclases, glycosyltransferases, transporters, and regulators. The expansion and clustering of the CYP450 superfamily are enough to highlight the important role of *G. lucidum* in secondary metabolism.

An intriguing finding was reported by the comparison of seven *Ganoderma* genomes, revealing a diverse array of terpene metabolism genes and secreted proteins, including CAZymes, lipases, proteases, and SSPs. Notably, *G. lucidum* and *G. lingzhi* carry the fewest terpene-related gene clusters in the group, whereas *G. australe* contains 31 terpene clusters among roughly 80 putative biosynthetic gene clusters [35]. The chemical diversity of *Ganoderma* species therefore depends on extensive CYP450-driven post-modification of lanosterol backbone.

## 3. Unraveling the Biosynthetic Gene Network of GTs via Multi-Omics

GTs biosynthesis is controlled by a complex gene network that integrates terpene backbone enzymes, CYP450, transcription factors, and post-translational regulatory components. Multi-omics approaches have greatly accelerated the dissection of GTs biosynthetic network from genome to metabolite. Integrated analysis of Illumina and PacBio sequencing platforms generated a golden annotation comprising 14,147 high-confidence genes. Within this annotation, 1272 polycistronic genes were identified, which encode 2815 protein-coding genes. These 1258 genes (44.69%) were enriched in F-box-like and P450 domains [38]. The importance of high-quality genomic annotations in studying the GTs biosynthesis genes, especially CYP450, is evident from the manual correction of the mis-split P450 gene. Zhu et al. (2015) [39] constructed a chromosomal-level genomic map of *G. sinense* employing complete genome sequencing, DNA methylation patterns and small RNA transcriptome data. This provided valuable insights into the genomic structure concerning secondary metabolism and defense responses. Together, these genomic resources enhance our understanding of the molecular mechanisms governing secondary metabolism and its regulatory circuits in *G. lucidum*.

Large-scale gene discovery for GTs biosynthesis has been greatly accelerated by transcriptomic profiling. A combined transcriptome and metabolome analysis across developmental stages of *G. lucidum* identified 5332 differentially expressed genes (DEGs) in the comparison of mycelium vs. primordia, and 4822 DEGs in the comparison of mycelium vs. fruiting bodies, with 3369 overlapping between the two comparisons. These DEGs were predominantly enriched in biological processes, including flavonoid biosynthesis, jasmonic acid-mediated signal pathway, electron transport chain activity, and secondary metabolite biosynthesis [40]. Similarly, in the transcriptome and targeted metabolomics analyses across different stages of *G. lucidum*, the targeted DEGs identified 699 metabolites, including 112 terpenoids. An integrative correlation analysis further associated the expression of six transcription factors and two CYP450 genes with the accumulation of four triterpenoid components [36]. Beyond developmental regulation, transcriptomic profiling has also been deployed to dissect geographic and elicitor-induced variations in GTs biosynthesis. Samples from six distinct cultivation regions have identified 34 CYP450 genes co-expressed with *LS*. Among these, 19 genes exhibited the positive correlation with GTs accumulation [41]. Transcriptome analysis of methyl jasmonate (MeJA)-exposed *G. lucidum* identified 103 differentially expressed transcription factors across 22 families. Correlation analysis showed that the expression levels of *GL23559* (MADS-box), *GL26472* (HTH), and *GL31187* (HMG) were positively correlated with total triterpene content, while *GL25628* (fungal-specific) and *GL26980* (PHD) correlated negatively [42]. Furthermore, in the transcriptomic response to exogenous sodium nitroprusside (SNP), the expressions of 12 genes within the terpenoid backbone pathway, including *HADH*, *FPS*, *DXS*, *PMVK*, *ACAT*, and *SQLE*, were significantly upregulated, whereas 8 genes (such as *HMGS*) were downregulated [17]. Transcriptomic and metabolomic profiling under heat stress also has revealed stage-specific metabolic reprogramming in *G. lucidum*. In the post-stress stage, upregulated genes were primarily enriched in stress response pathways, including the TCA cycle, oxidative phosphorylation and ABC transporter genes. While in the recovery stage, the upregulated genes were enriched in pathways related to lipid decomposition, fatty acid β-oxidation and terpene skeleton biosynthesis. These findings suggest that heat stress promotes GTs accumulation through the sequential activation of distinct metabolic modules [14].

A recent multi-omics framework integrated metabolomics, transcriptomics and proteomics data from *G. lucidum* strains across six developmental stages. Simultaneously, weighted gene co-expression network analysis (WGCNA) identified multiple modules associated with GTs biosynthesis. The results revealed the central transcription factor Zn_2_Cys_6__61 and facilitated the identification of differentially expressed CYP450 candidate genes potentially involved in the structural modification of GTs [28]. Furthermore, acetylomic analysis demonstrated that *Glsirt1*-silencing resulted in hyperacetylation of numerous proteins involved in energy metabolism and stress responses. Among these, catalase (*Gl*CAT) was identified as a direct deacetylation target of *Gl*sirt1, notably, acetylation of *Gl*CAT (at K123 site) increases its activity, whereas *Gl*sirt1-mediated deacetylation inhibits it, so *Gl*sirt1 promotes ROS accumulation and GTs biosynthesis by keeping *Gl*CAT in a deacetylated, low-activity state [43]. This establishes a mechanistic link between the post-translational modifications of proteins and GTs biosynthesis. This is therefore an expansion of the regulatory framework from transcriptional and metabolic networks to epigenetic modulation. In conclusion, these findings not only enhance our understanding of GTs biosynthetic regulation but also provide actionable targets for improving yield via metabolic engineering.

## 4. Signal Transduction Networks: From Independent Pathways to Crosstalk

Environmental stressors and exogenous elicitors essentially operate by triggering complex intracellular signal cascades, which ultimately convert external stimuli into transcriptional responses. In the past decade, efforts have been made to elucidate the mechanisms underlying elicitation action, enabling the preliminary mapping of the signal transduction network controlling GTs biosynthesis in *G. lucidum*. In this part, we mainly take these three signal pathways (ROS, Ca^2+^, NO) as examples to elaborate on the regulatory mechanisms in GTs biosynthesis and their crosstalk.

### 4.1. ROS Signal

The GTs biosynthesis is finely regulated by diverse environmental stresses and signal molecules. Among these signal elements, ROS function as important signal molecules which transfer extracellular stimuli into intracellular metabolic reprogramming. This ROS-mediated regulatory system involves multiple levels, including the formation and maintenance of ROS homeostasis, ROS signal perception and transduction into downstream biosynthetic pathways, as well as interconnection with other signal molecules [4]. In *G. lucidum*, ROS production is enzymatically catalyzed by a certain enzyme system, but not stochastic, and results from the activation of both NADPH oxidase and the mitochondrial electron transport chain (ETC) [44,45].

The family of NADPH oxidases (Nox) (*NoxA*, *NoxB*, and *NoxR*) is considered the primary enzymatic source of ROS elicited by the environmental stimuli in *G. lucidum*. Cu^2+^ treatment strongly induced the transcription levels of Nox-encoding genes. In parallel with that, the ROS burst exhibited strong spatiotemporal and dose-dependent correlation with the accompanying increase in the content of GTs mycelial branching density, and intracellular Ca^2+^ levels [16]. The use of the Nox inhibitors or their silencing effectively suppressed the ROS generation, and the subsequent GTs accumulation induced by Cu^2+^ or MeJA treatment [16,44]. Moreover, under heat stress conditions, *Nox*-silenced strains displayed significantly decreased ROS levels and GTs accumulation compared to the wild-type, thereby confirming Nox as an important enzyme responsible for the ROS burst caused by heat stress [46]. These observations collectively position Nox as a central transducer linking the environmental sensing to metabolic responses in GTs biosynthesis.

A critically important relationship exists between ROS production and mitochondrial function. In the physiological metabolism of *G. lucidum*, perturbations in key metabolic nodes cause an increase in the flow of metabolic flux towards maintaining basal energy supply. This redirection coincides with increased mitochondrial ROS levels, which cause the activation of the defensive secondary metabolism. Treatment with salicylic acid (SA, 100–400 μM) inhibited mitochondrial Complex III activity from 51% to 75%, resulting in the ROS burst and GTs overproduction. *Nox*-silencing could partially attenuate these SA-induced phenomena, implying that SA activates both Nox and mitochondrial complex III for ROS generation [47]. Supporting evidence comes from other studies on mitochondrial alternative oxidase (AOX), where *AOX*-silencing increased intracellular ROS levels by 1.75- to 1.93-fold and concurrently promoted GTs content by 42–44% [18]. In addition, *got1*-silencing raised intracellular NADH levels by 2.7-fold, accompanied by a concomitant reduction of about 67% in the NAD^+^/NADH ratio. This alteration was also observed to be associated with a 1.5-fold increase in mitochondrial ROS levels and a 40% elevation in GTs content [48]. All the observations demonstrate that mitochondria plays a crucial role in integrating metabolic status and stress signals to produce functional ROS, potentially linking ROS production with GTs metabolism. However, such a positive effect operates only within a certain range of ROS flux.

The ROS homeostasis is maintained through an intricate antioxidant defense system and their post-translational modifications within the cell. Silencing of glutathione peroxidase (*GPx*) increased intracellular H_2_O_2_ levels but markedly reduced GTs content. Mechanistic studies indicated that excessive ROS caused by *GPx* deficiency suppressed Ca^2+^ signal, which subsequently decreased GTs biosynthesis [49]. The deacetylase *Gl*sirt1 was shown to deacetylate and inhibit *Gl*CAT activity, while also promoting ROS accumulation and GTs biosynthesis. Subsequent research identified *Gl*CAT as the principal downstream effector mediating *Gl*sirt1-induced ROS production [43]. These offer a new perspective on the role of post-translational modifications in the ROS-mediated regulation of secondary metabolism. Liu et al. (2022) [50] further demonstrated that silencing of S-nitrosoglutathione reductase (GSNOR) triggered hyper-S-nitrosylation of CAT, which led to an increase in CAT activity and reduction in ROS levels, ultimately suppressing heat stress-induced GTs accumulation. These findings define a “dual switch” for CAT activity, mediated by deacetylation (via *Gl*sirt1) and S-nitrosylation (whose abundance is counteracted by GSNOR), which exert antagonistic regulation on CAT activity through two distinct post-translational modifications, respectively. Additionally, heat stress also triggered the generation of endogenous hydrogen sulfide (H_2_S), which persulfidated thioredoxin 1 (Trx1) at the Cys31 and Cys34, thereby enhancing its catalytic activity. The activated Trx1 subsequently mitigated excessive ROS accumulation, establishing a negative feedback loop for GTs biosynthesis [51].

Consequently, ROS function not only as a key messenger that initiates defense-related secondary metabolism in response to environmental stress in *G. lucidum*, but also as a central hub coupling upstream stress perception and downstream transcriptional reprogramming. Maintaining ROS homeostasis is therefore critical for efficient GTs biosynthesis.

### 4.2. Ca^2+^ Signal

The Ca^2+^ signal acts as a multifunctional signal mediator in cellular physiology, playing a crucial role in regulating various biological processes, such as cell division, differentiation, stress responses, and secondary metabolism [52,53]. The Ca^2+^ signal is increasingly recognized as a crucial regulatory hub within the network that controls GTs biosynthesis [4]. The cytoplasmic free Ca^2+^ concentration ([Ca^2+^]_cyt_) in fungi is very low (50–100 nM) under the resting state of cells. Upon perception of environmental stresses or exogenous elicitors, [Ca^2+^]_cyt_ spikes rapidly to 10^−5^ M, generating characteristic Ca^2+^ transients [54]. These transients primarily result from extracellular Ca^2+^ influx mediated by plasma membrane calcium channels and Ca^2+^ release from intracellular pools [55]. Notably, Xu et al. (2012) [56] systematically revealed the enhancing effect of exogenous Ca^2+^ on the GTs accumulation, particularly noting the varying degrees of increase in the amounts of monomers of ganoderic acid-Mk (GA-Mk), GA-T, GA-S, and GA-Me. The Ca^2+^ addition not only increased the expressions of key enzyme genes (*hmgr*, *ls*, *sqs*), but also upregulated the Ca^2+^ signal components (*cam* and *cna*). Beyond direct elicitor perception, Ca^2+^ signal can also be activated indirectly through ion exchange mechanisms. Electrochemical gradients generated by ATPases and cation exchangers drive passive Ca^2+^ reflux. The Na^+^/Ca^2+^ exchanger and Mn^2+^/Ca^2+^ P-type ATPases (e.g., *PMR1*) can respond to the exogenous additions of Na^+^ or Mn^2+^ and regulate Ca^2+^ influx or alter its intracellular distribution, thus modulating GTs biosynthesis [57,58].

Environmental stresses function as endogenous triggers that activate Ca^2+^ signal and induce the accumulation of secondary metabolites. Cu^2+^ and Ca^2+^ induction effectively elevate [Ca^2+^]_cyt_ levels and stimulate GTs biosynthesis [16,55]. Specifically, silencing of the calcium-permeable channel gene (*cch*) and hosphoinositide-specific phospholipase (*plc*) significantly reduced heat shock protein (HSP) expression and GTs accumulation by 50–70%. This finding demonstrates that these channel proteins in Ca^2+^ signal serve as critical gateways for transmitting heat stress signals into the cell. Furthermore, abscisic acid (ABA) promoted GTs accumulation by activating [Ca^2+^]_cyt_ and regulating the expression of relevant channel genes, including *cch1*, calcium transporting ATPase, calcium-dependent mitochondrial carrier protein, vacuolar calcium ion transporter, and calcium/calmodulin-dependent protein kinase *cmkB* [59]. While the transient elevation of [Ca^2+^]_cyt_ levels is not inherently informative, the specificity of the Ca^2+^ signal is imparted by a series of conserved downstream decoders, including Ca^2+^-binding proteins and their effectors. As exemplified by the finding that silencing of Calmodulin (*CaM*) suppressed GTs accumulation and mycelial growth induced by Ca^2+^ or heat stress [8]. Upon Ca^2+^ binding to its EF-hand motifs, CAM experiences a conformational change that activates downstream targets, with calcineurin being one of the most significant effectors. During exogenous Na^+^-induced Ca^2+^ influx, the calcineurin signal is activated and subsequently modulates GTs biosynthesis by regulating the expression levels of biosynthetic genes and Ca^2+^ sensors. This highlights the role of calcineurin as a necessary node mediator that bridges Ca^2+^ signal and GTs metabolism [57]. These findings indicate that Ca^2+^ signal serves as a crucial link between environmental stimuli and GTs accumulation, providing a rationale for developing a transduction pathway to increase GTs production in *G. lucidum*.

### 4.3. NO Signal

NO is a gaseous free radical signal molecule that is involved in different physiological processes in fungi, including development, stress responses, and metabolism [60]. There are two main ways for NO biosynthesis: the arginine oxidation pathway proceeding via nitric oxide synthase (NOS), and the nitrite reduction pathway occurring through nitrate reductase (NR) and nitrite reductase [61]. In *G. lucidum*, the NR-dependent pathway acts as the major manner for NO production [62]. Functional evidence supports that *NR*-silencing or applying an NR inhibitor completely abolishes NO production and eliminates the GTs enhancement induced by MeJA or heat stress, which establishes NR as a key enzymatic hub in stress-responsive NO generation [8,62]. Furthermore, *G. lucidum* can use nitrate as its sole nitrogen source. In the absence of ammonia or glutamine, which are the preferred nitrogen sources, nitrate is assimilated, resulting in the production of NO through the NR-catalyzed step. Mycelia grown on nitrate exhibited approximately 2.7-fold higher levels of NO and about 2.3-fold greater NR activity compared to those grown on ammonia [21].

The regulation of NO in GTs biosynthesis exhibits a characteristic bidirectional regulatory feature that is both dose-dependent and condition-dependent, which is determined by the cell’s physiological status, NO concentration threshold, and its crosstalk with parallel signals. Exogenous treatment with SNP has demonstrated that NO could enhance GTs accumulation while simultaneously suppressing mycelial growth. Transcriptomic studies have further revealed that repression of growth by NO is caused by the inhibition of carbohydrate metabolism, shifting carbon flux from primary to secondary metabolism for GTs production [17]. On the other hand, NO performs a negative regulatory role during heat stress. Although heat stress induced GTs accumulation and triggered a significant elevation in NO levels, removal of NO through scavenging with using c-PTIO (an NO scavenger) further increased GTs content by 25%, whereas SNP decreased GTs content by 30% [8]. This contradictory response reveals that stress-induced NO functions as a built-in limiter that curbs excessive activation of secondary metabolism, thereby preserving the delicate balance between stress responses and metabolic homeostasis. Moreover, a similar negative regulatory mechanism has also been observed in nitrogen metabolism [21].

The effect of NO on protein regulation occurs through three mechanisms: the soluble guanylate cyclase (sGC)-mediated cGMP pathway, direct coordination with metalloprotein active sites, and S-nitrosylation of specific cysteine residues [63]. In *G. lucidum*, S-nitrosylation plays an important role as the post-translational modification for NO-dependent regulation of GTs biosynthesis. For example, NO decreased aconitase (Acon) activity via S-nitrosylation at Cys-594, causing a 60% decrease in mitochondrial ROS production and finally attenuating heat-induced GTs accumulation [64]. GSNOR is responsible for modulating ROS levels under heat stress through modulating the S-nitrosylation status of *Gl*CAT; three novel S-nitrosylation sites on the CAT were identified, including Cys401, Cys642 and Cys653, of which the mutation of C401 to serine (C401S) decreased the responsiveness of CAT to GSNO [50]. Furthermore, the regulatory role of NO in terpenoid synthesis has been thoroughly demonstrated in various plant systems, such as *Salvia miltiorrhiza*, *Betula platyphylla*, and *Catharanthus roseus* [65,66,67]. These findings indicate that NO is a conserved signal molecule regulating terpenoid synthesis, with a shared evolutionary origin and functional module across fungi and plants.

### 4.4. Crosstalk of Different Signals

**Figure 2:**
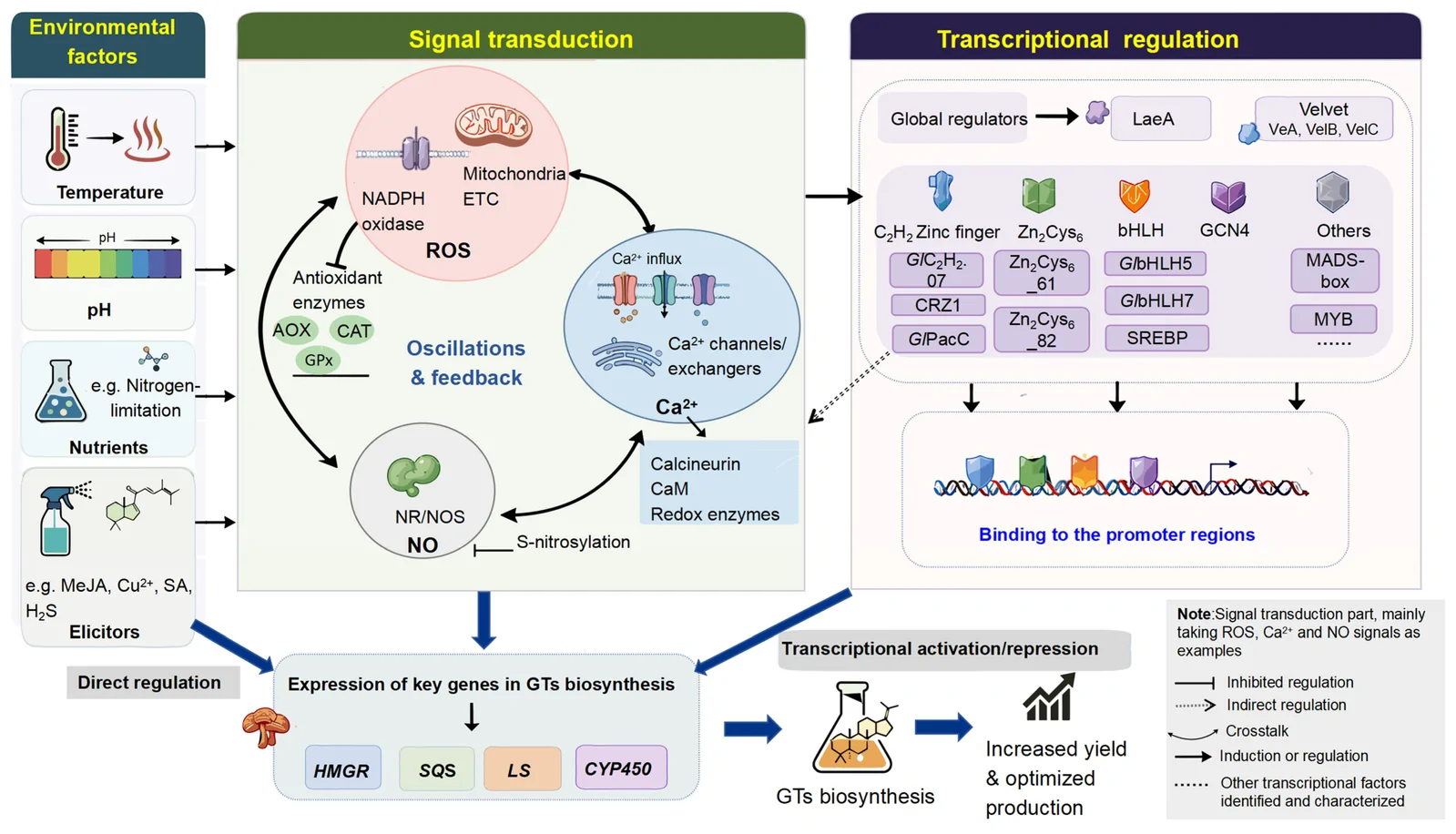
Overview of environmental elicitor-triggered signal transduction and transcriptional regulatory networks governing GTs biosynthesis in *G. lucidum*. (Heat stress: 42 °C) [55]; pH: two stage (3 to 4.5) [68]; Nitrogen-limitation: glutamine (60 to 3 mM) [15], nitrate (30 mM) [21]; MeJA: 50–254 μM [69]; Cu^2+^: 2–5 mM [16]; SA: 100 μM [47]; H_2_S: 90 μM (NaHS) [70].

In the secondary metabolism network of *G. lucidum*, ROS, Ca^2+^ and NO signals serve as discrete signal nodes. However, they also form a tightly interconnected ternary regulatory center through frequent and complex bidirectional interactions. This intricate interplay allows cells to respond with precision, dynamism, and, at times, in opposing manners (such as the bidirectional regulation of NO) to environmental stresses [4,8,62] (Figure 2).

Among the interconnections within the GTs regulatory network, the interaction between ROS and Ca^2+^ exemplifies a particularly prominent bidirectional crosstalk that forms an oscillation loop. Environmental stressors such as Cu^2+^, SA or MeJA activate Nox or the mitochondrial ETC and trigger an intracellular ROS burst [16,44,47]. Elevated ROS then activate plasma membrane Ca^2+^ channels (e.g., *cch1*), continuously promoting extracellular Ca^2+^ influx and [Ca^2+^]_cyt_ concentration. This cascade of Ca^2+^ mobilization is essential for the generation of stress-induced Ca^2+^ signals [55,71]. Under Cu^2+^ stress, the ROS scavenger N-acetylcysteine (NAC) suppressed the [Ca^2+^]_cyt_ increase by 70% and prevented GTs accumulation [16]. Similarly, in the MeJA-elicited cascade, Nox-derived ROS production occurred prior to and was necessary for the activation of Ca^2+^ signal, which subsequently engaged downstream transcriptional regulation [62,71]. All these indicate that ROS typically function upstream or in parallel to the Ca^2+^ signal, acting as the initial responder to environmental stresses.

The crosstalk between ROS and Ca^2+^ is not limited to a cascade but also includes negative feedback regulatory loops, where Ca^2+^ controls ROS overproduction. This results in the prevention of oxidative damage and maintenance of cellular homeostasis. Specifically, Cu^2+^-induced GTs biosynthesis was critically reliant on the ROS burst, which subsequently triggered Ca^2+^ influx. In turn, the Ca^2+^ signal contributed to the increase in activities of SOD and CAT, while decreasing ROS levels and GTs production. This observation directly proves the function of the Ca^2+^ signal in a negative feedback way [16]. In the ABA-mediated Ca^2+^ signal in the process of GTs biosynthesis, activation of the antioxidant defense system could be seen in the higher levels of SOD and APX, as well as increased GSH content, while the levels of H_2_O_2_ and MDA were considerably lower [59]. This finding shows the importance of Ca^2+^ signal in coordinating the defensive response. The negative feedback system is responsible for the safety threshold for GTs biosynthesis. When ROS levels exceed the optimal signal range, such as ROS overaccumulation caused by *GPx*-silencing, a higher Ca^2+^ signal reduces the ROS levels via increasing the antioxidant capacities [49]. It can explain the decrease in GTs biosynthesis when there are high ROS levels under GPx deficiency. Functionally, the oscillatory loop amplifies the primary ROS to Ca^2+^ cue, while the concurrent negative feedback prevents stress overload, thereby ensuring that ROS levels remain within the optimal range for GTs production.

The interplay between NO and ROS is highly context-dependent, leading to opposite regulatory effects under different stress conditions. In the presence of MeJA, NO was involved in increasing ROS levels by promoting the NR activity, which served as a pro-synthesis signal for GTs accumulation [62]. Conversely, under heat stress, NO reduced the amount of mitochondrial ROS by roughly 60% through the inhibition of Acon activity, which in turn suppressed GTs accumulation. This divergent behavior arisen from the target specificity of NO-mediated S-nitrosylation [64]. The identical NO signal can either activate or inhibit ROS levels, contingent upon the target enzymes involved (NR, Acon or CAT) and the specific modification sites engaged [43,62,64]. Such source- and spatiotemporal-dependent functional bifurcation of ROS illustrates how cells utilize NO-mediated redox modifications to finely regulate GTs metabolism in response to distinct environmental stimuli.

A bidirectional positive regulatory relationship exists between NO and Ca^2+^ signal under heat stress, forming a mutually reinforcing positive feedback loop. The application of the NO scavenger cPTIO, the Ca^2+^ chelator EGTA, *CaM*- and NR-silencing all attenuated the generation of this reciprocal signal, indicating that the activities of NR or *cch1*/PLC are subject to cross-regulation by the counterpart signal [8,55]. This cross-regulation is a positive feedback amplification loop where both processes amplify the cellular response to heat stress in a cooperative manner. However, NO and Ca^2+^ play distinct roles in regulating GTs accumulation. In particular, in terms of the hierarchy of heat stress-induced signal, Ca^2+^ plays a more crucial upstream role, while NO is a secondary modulator [8]. The major physiological function of elevated NO signal is not to directly drive metabolic flux; instead, it acts as a negative feedback mechanism that mitigates metabolic disturbances caused by excessive heat stress and helps maintain cell homeostasis. This cooperative yet hierarchically dominant regulatory pattern illustrates the advanced regulation that is exhibited by *G. lucidum* under stress conditions.

## 5. Key Transcription Factors Governing GTs Biosynthesis

In signal networks, transcription factors are key executors that integrate signals and reprogram target gene expression [72]. They precisely regulate the accumulation of secondary metabolites by recognizing and binding to cis-acting elements within target gene promoters, thereby activating or inhibiting the transcription of genes involved in secondary metabolic pathways [36]. In recent years, with the completion of the genome sequencing of *G. lucidum*, along with advancements in multi-omics profiling and signal network dissection, an increasing number of transcription factors have been identified and functionally verified in the regulation of GTs biosynthesis in *G. lucidum*.

### 5.1. Global Transcription Factors

Global transcription factors coordinate secondary metabolism by simultaneously modulating multiple genes across distinct biosynthetic pathways. Notable examples include LaeA and Velvet family proteins, which have been identified as essential regulators of triterpene biosynthesis in ascomycetes and basidiomycetes.

LaeA (loss of *aflR* expression-A) is a methyltransferase that differentially regulates expression of various secondary metabolite gene clusters in fungi [73]. Luo et al. (2022) [74] first identified and characterized the regulatory role of LaeA in the GTs biosynthesis in *G. lucidum*. The LaeA protein (encoded by *gl27879*) comprises 375 amino acids, with the S-adenosylmethionine-dependent methyltransferase domain located between residues 115 and 295. Following the knockout of *LaeA* using a CRISPR/Cas9 dual-sgRNA strategy, the contents of total GTs, GA-T, and GA-Me were all significantly reduced, along with a marked transcript downregulation of *sqs* and *ls*. This indicates that LaeA regulates GTs flux through the transcriptional control of key biosynthetic enzymes. The function of LaeA in terpene metabolism has been well established in Ascomycetes. In *Fusarium graminearum*, *Fg*LaeA acts as a positive regulator of mycotoxin biosynthesis, including trichothecenes and zearalenone. According to transcriptomic profiling, approximately 70% of the gene cluster members were found to be differentially expressed in the *ΔFgLaeA* strain [75]. Recent studies in *Trichoderma hypoxylon* have revealed a contrasting scenario. The single knockout of *ThLaeA* did not exert a major influence on secondary metabolite production. On the contrary, the *ΔThtri5* mutant that was defective in trichodiene synthase, the *ΔthLaeA* mutation could restore the biosynthesis of key terpene compounds that were inhibited by *ΔThtri5*. This result indicates that *Th*LaeA functions as both an activator and a conditional inhibitor within a specific genetic framework. This dual role contributes to metabolic homeostasis by balancing Thtri5-dependent disturbances [76].

The Velvet protein family represents a highly conserved group of global regulatory proteins in fungi and comprises four core members: VeA (Velvet), VelB (Velvet like B), VelC (Velvet like C), and VosA (viability of spores A). These proteins contain a fungal-specific Velvet domain [77], and are essential for controlling sexual reproduction, asexual reproduction, and secondary metabolite synthesis in fungi [78]. In *G. lucidum*, Meng et al. (2022) [40] identified three genes encoding Velvet family proteins: VeA (*GL19230*), VelB (*GL21819*), and VelC (*GL23472*). The RNAi-mediated knockdown of *VeA* or *VelB* reduced GTs content via decreasing the expression of key biosynthesis-related genes, including *HMGR*, *SQS*, and *OSC*. Moreover, the expression of distinct Velvet family proteins exhibited different levels across various developmental stages. Therefore, Velvet family proteins serve as critical positive regulators of both development and GTs biosynthesis in *G. lucidum*. The LaeA and velvet protein multimeric complexes play crucial roles in controlling secondary metabolism, light signal response, and developmental regulation in fungi [73,79]. In an engineered strain of *G. lucidum*, overexpression of VeA-VelB complex and LaeA enhanced secondary metabolism and promoted high yields of meroterpenoids. In addition, by reconstructing the terpene biosynthesis pathway and optimizing the accumulation of the triterpene precursor 2,3-oxidosqualene, the production of oleanolic acid and ursolic acid was markedly increased [80]. Thus, the manipulation of global regulators provides efficient ways sustainable production of high-value triterpenoids in fungal cell factories.

### 5.2. Cys_2_His_2_-Type Zinc Finger Family

In the genome of *G. lucidum*, the C_2_H_2_-type zinc finger family represents one of the most abundant families of transcription factors. The zf-C_2_H_2_ DNA-binding domain itself is evolutionarily conserved, and every member carries at least one such domain. However, a conserved transcription factor does not guarantee a conserved regulon; the target genes it activates or represses can diverge substantially across species [81]. Treatment with MeJA significantly increased the GTs accumulation and activated 103 differentially expressed transcription factors (95 upregulated and 8 downregulated) across 22 families, with the C_2_H_2_ family being the most highly represented, comprising 15 members in total [42]. Throughout different developmental stages of *G. lucidum*, 53 potential transcription factor families were identified. Among these, the C_2_H_2_, Zn_2_Cys_6_, and Zf-MYND families exhibited distinct stage-specific patterns. Specifically, the C_2_H_2_ family displayed the highest transcriptional levels during the mycelium stage with 15 DEGs upregulated, implicating its role as a key regulator of GTs metabolism during early development [40].

The C_2_H_2_-type zinc finger protein *Gl*C_2_H_2_.07 was identified and localized in the cell nucleus in *G. lucidum*. Overexpression of *Gl*C_2_H_2_.07 significantly promoted GTs accumulation, confirming its positive regulatory properties [82]. Transcriptomic profiling further indicated that the regulatory targets of *Gl*C_2_H_2_.07 included key enzymes of MVA pathway, including MPK, FPS and LS, along with several cytochrome P450 genes (*CYP5035M2*, *CYP5144C41*, *CYP5144C54*, *CYP5147B1*). This suggests that *Gl*C_2_H_2_.07 coordinates the entire GTs network by integrating primary carbon metabolism and regulating the steps from precursor supply to structural diversification. Calcineurin-responsive transcription factor 1 (CRZ1), another C_2_H_2_-type zinc finger transcription factor, operates downstream of the CaM-calcineurin signal pathway. Upon Ca^2+^ stimulation, CRZ1 is dephosphorylated by calcineurin and rapidly translocated to the nucleus, where it binds to calcineurin-dependent response elements (CRDEs) in target gene promoters to regulate transcription [83]. Li and Zhong (2020) [84] identified two CRZ1 homologs in *G. lucidum*: *Gl*CRZ1 (GL25464) and *Gl*CRZ2 (GL26097). Among these, *Gl*CRZ1 primarily transmitted the Ca^2+^ signal to activate genes involved in GTs biosynthesis, while *Gl*CRZ2 exerted a broader role in maintaining cell growth and metabolic homeostasis, showing the functional differentiation of CRZ1 family transcription factors in the regulation of secondary metabolism. Similarly, it has been reported that the transcription level of CRZ1 significantly increased during sodium acetate-induced GTs biosynthesis [85]. All these findings demonstrate that exogenous elicitors can modulate intracellular Ca^2+^ signal to fine-tune GTs biosynthesis through specific transcription factors such as *Gl*CRZ1 [19,84]. *Gl*PacC, a homolog of the pH-responsive transcription factor PacC containing three C_2_H_2_-type zinc finger domains, was successfully identified in *G. lucidum*. *Gl*PacC-silencing attenuated pH-dependent growth adaptation while concurrently upregulating the transcriptions of *SQS* and *LS* and increasing intermediate metabolite levels. Moreover, such silencing led to aberrant mycelial growth, consequently inhibiting the formation of primordia and fruiting bodies [86]. This functional characterization reveals that *Gl*PacC plays a pleiotropic role in both secondary metabolism and morphological differentiation.

C_2_H_2_-type zinc finger-containing transcription factors are responsible for terpene biosynthesis in different fungi and plants with both conserved and lineage-specific regulatory characteristics. In *Aspergillus nidulans*, *mtfA* acts as a regulator of the production of the mycotoxin sterigmatocystin (ST) via controlling the expression of *AflR*, which is a pathway-specific activator of the ST gene cluster [87]. In *Aquilaria sinensis*, AsZFP9 interacts with and activates the transcription of two key genes in sesquiterpene biosynthesis, *AsHMGR1* and *AsTPS1* [88]. In *Artemisia annua*, AaZFP1 functions as a transcriptional activator by interacting with the promoter of *AaIPPI1* and upregulating the transcription process leading to artemisinin biosynthesis [89]. Despite these mechanistic similarities, such as direct DNA binding and transcriptional activation, there are substantial differences in their target genes and associated pathways, which highlights the evolutionary adaptability of this transcription factor family within specialized metabolic systems.

### 5.3. Zn_2_Cys_6_ Family

As one of the largest transcription factor families specific to fungi, the Zn_2_Cys_6_ family is essential in the regulation of various biological processes, such as carbohydrate metabolism, amino acid biosynthesis, the stress response, and secondary metabolism [90]. Zn_2_Cys_6__61 has been shown to be a major positive regulator in the GTs biosynthetic network of *G. lucidum*. It binds directly to the G-box element within the *SQS* promoter, which activates the *SQS* transcription and redirects metabolic flux from the sterol biosynthesis to the GTs production. This finding makes Zn_2_Cys_6__61 an excellent candidate for metabolic engineering modifications [28]. Zn_2_Cys_6__82 is another positively regulating transcription factor within the Zn_2_Cys_6_ family in *G. lucidum*. During both the mycelium and primordium stages, overexpression of Zn_2_Cys_6__82 increased the total GTs content and highly induced the expression levels of *SQS* and *HMGR*. Moreover, distinct changes in GA monomers were observed in *Zn*_2_*Cys*_6_*_82*-overexpressing and -silenced strains, indicating that Zn_2_Cys_6__82 may selectively regulate different branches of the triterpene pathway [91]. These observations further confirm that various members of the Zn_2_Cys_6_ family may collaborative in the transcriptional control network of GTs biosynthesis.

Zn_2_Cys_6_ transcription factors have been implicated in terpenoid biotransformation and other specialized metabolic processes across various fungi. In *Penicillium digitatum*, PdTP1, which contains a GAL_4_-like Zn_2_Cys_6_ DNA-binding domain and a fungal_trans domain, facilitated the biotransformation of limonene and enhanced α-terpineol production by regulating genes associated with terpene metabolism. This study provided the first evidence that Zn_2_Cys_6_ transcription factors can regulate monoterpenoid biotransformation in fungi [92]. In *Cordyceps militaris*, the putative Zn_2_Cys_6_ transcription factor *Cmcrf1* was confirmed to participate in carotenoid biosynthesis; this was evidenced by a significant reduction in carotenoid production in the *ΔCmcrf1* mutant, which also failed to produce fruiting bodies [93]. Additionally, a sequence in the C-terminal half of the SUT1 protein shows significant homology to the Zn_2_Cys_6_ binuclear cluster DNA-binding domain [94]. SUT1 promoted sterol uptake by coordinately upregulating the expression of its downstream target genes *Aus1* and *Dan1*, which functioned synergistically in this process [95]. This transcription factor family also orchestrated the regulatory network that involved fungal fatty acid synthesis and aflatoxin production [96,97].

### 5.4. Basci Helix-Loop-Helix (bHLH) Family

bHLH transcription factors represent a prominent family of fungal regulatory proteins that exert pivotal roles in vegetative growth, differentiation, and secondary metabolism [98]. Li et al. (2026) [99] systematically identified 11 *Gl*bHLH members in *G. lucidum*. Among these, *Gl*bHLH5 and *Gl*bHLH7 consistently exhibited the highest expression levels throughout all developmental stages and demonstrated a strong correlation with key genes involved in the MVA pathway. Functionally, *Gl*bHLH5 directly binds to and activates the P2 region of *LS* gene promoter, while *Gl*bHLH7 binds to the promoters of the *SE* and *SQS* genes. The bHLH promoter region contains multiple cis-acting elements responsive to abiotic stress and phytohormones (e.g., MeJA, ABA, SA), which are associated with fungal growth, secondary metabolism and the stress response [100]. Treatment with MeJA concurrently induced the upregulation of *GlbHLH5* and the key enzyme genes *HMGR*, *SQS*, and *LS*. Furthermore, overexpression of *GlbHLH5* increased GTs content by 21–45% and upregulated the transcript levels of these three key enzymes by 2.6- to 3.2-fold, thereby confirming its role as a positive regulator of GTs biosynthesis in *G. lucidum* [101].

The transcription factor SREBP is characterized by a conserved bHLH-Zip domain and plays a critical role in the regulation of cholesterol and fatty acid metabolism in mammals [102]. In *G. lingzhi*, the *SREBP* gene features a 2532 bp open reading frame encoding a protein with an N-terminal bHLH DNA-binding domain (residues 197–294 aa). Functional characterization through SREBP overexpression (OE::SREBP) demonstrated that the upregulation of genes was linked to sterol and triterpene biosynthesis, including 11 key enzymes in the MVA pathway (e.g., SQS, HMGS and MVK). Metabolomic profiling analysis has also shown the increased levels of intermediates, triterpenoids, and the total amount of CYP450 [23]. Such dual activation of precursor supply and downstream oxidative modifications makes SREBP a key regulator of GTs flux. Moreover, under heat stress, the phosphatidic acid-activated mTOR pathway promotes the processing of nuclear SREBP, which functions downstream of mTOR to drive the transcription of its target genes, including *hmgcs*, *mk*, *sqs* and *lss*, thus coupling stress perception to GTs biosynthesis [103].

### 5.5. General Control Nonderepressible 4 (GCN4) Transcription Factor

GCN4 is a central transcription factor belonging to the basic leucine zipper (bZIP) family. Under nitrogen-limited conditions, GCN4 activates and drives the expression of amino acid biosynthetic genes to compensate for nitrogen deficiency [104]. This nutrient-responsive function has recently been expanded to GTs biosynthesis, establishing GCN4 as a key interface between nitrogen availability and secondary metabolite production in *G. lucidum*.

In nitrogen-limited conditions, GCN4 binds to the promoters of antioxidant enzyme genes (*gr*, *gst2*, and *cat3*) and regulates the antioxidant system, reducing ROS levels and curtailing GTs overaccumulation. It has been demonstrated that GCN4 exerts negative regulation on GTs biosynthesis through ROS signal, maintaining a balance between defense activation and metabolic homeostasis [105]. This negative regulation is supplemented by an additional layer of control at the carbon metabolic level. GCN4 can directly bind to and activate the promoter of the mitochondrial pyruvate transporter gene *GlMPC1/2*, which eventually enhances pyruvate import into mitochondria and oxidative metabolism, ultimately modulating carbon flow distribution and inhibiting GTs biosynthesis [106]. Lian et al. (2022) [107] revealed that GCN4 targets the *AreA* promoter, while SKO1 enhances GCN4′s binding affinity by 14.2-fold through heterodimerization, thereby amplifying *AreA* transcriptional output. This finding further elucidated the hierarchical regulatory function of GCN4 in the nitrogen metabolic network. In this network, AreA performs dual roles in GTs biosynthesis. On the one hand, it directly activates the expression level of *fps* by binding to its promoter, while indirectly suppressing GTs biosynthesis through NO production during nitrate assimilation [21]. At another level of regulation, the GCN4 binds to the GBE motif in *SWI6B* promoter and activates its transcription. The resulting GCN4-Swi6B module promoted cell wall thickening and the accumulation of chitin and β-1,3-D-glucan under low-nitrogen conditions [108]. Collectively, GCN4 is a multifaceted regulator of stress adaptation, metabolic balance and developmental processes, establishing it as a key target for metabolic engineering to enhance GTs production.

Besides the well-established regulators outlined above, numerous additional transcription factors from various families also participate in the GTs biosynthesis in *G. lucidum*. These include MADS-box, Homeobox, bZIP, APSES, and MYB family members, which execute their regulatory functions via following discrete mechanistic modes: direct modulation of MVA pathway genes, mediation of ROS/Ca^2+^/NO signals, responsiveness to environmental elicitors or stresses, and epigenetic modifications or non-canonical mechanisms [109,110,111]. These transcription factors integrate developmental stage-specific cues with environmental variations, thereby optimizing the transcriptional activity of MVA pathway genes and downstream tailoring enzymes to maximize GTs accumulation. A detailed summary of their individual functions and key effects is provided in Table 1.

## 6. Engineering Strategies for Enhancing GTs Biosynthesis

**Figure 3:**
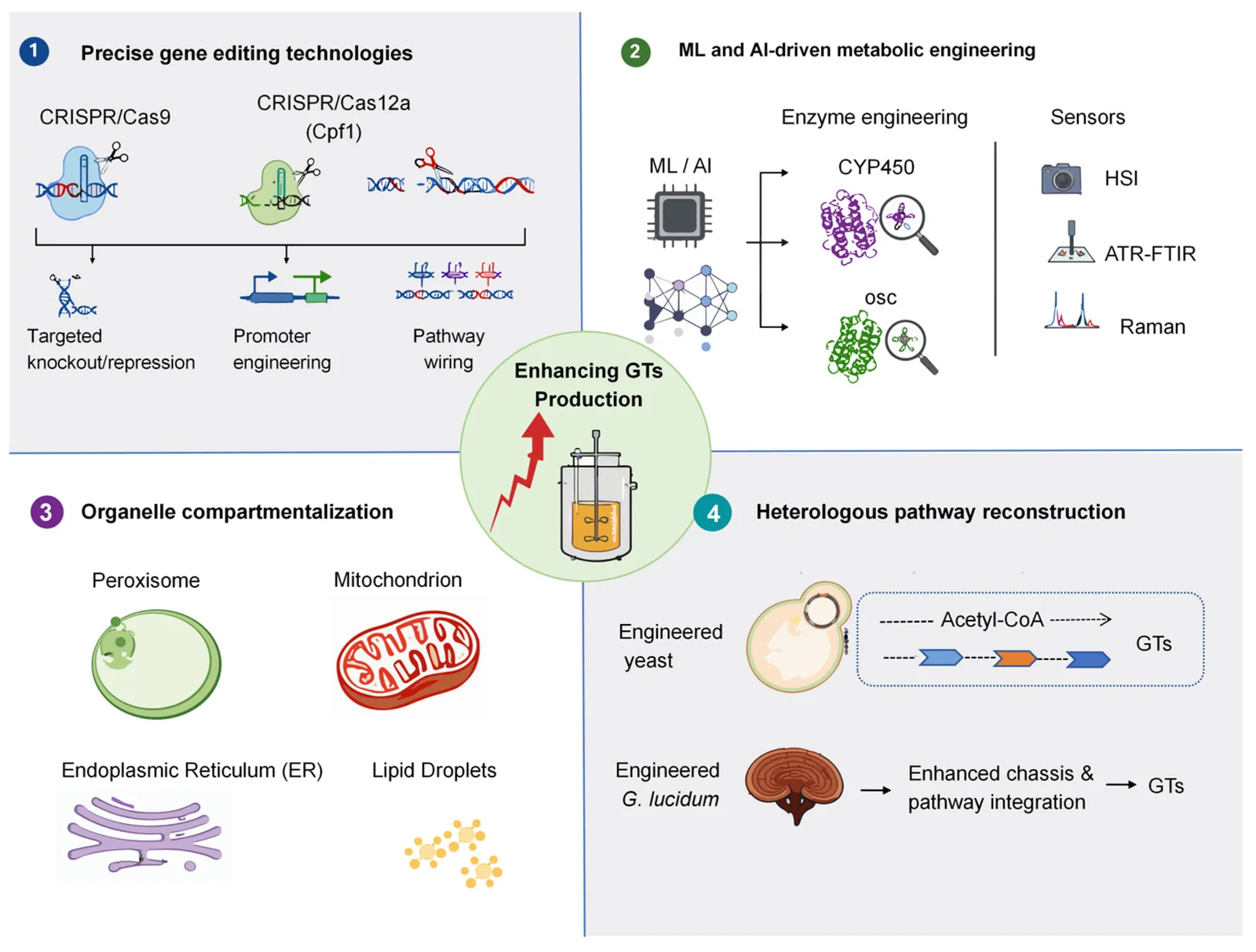
Multi-dimensional engineering strategies and enabling technologies for enhancing GTs production.

Advancements in multi-omics profiling, signal transduction networks, and transcriptional regulation have progressively shifted GTs biosynthesis to predictive reconstruction and intelligent optimization. Recently, the rapid development of innovative technologies, such as CRISPR gene editing, ML-guided metabolic engineering, and organelle compartmentalization, has provided a multi-dimensional technical toolbox for addressing the GTs yield bottleneck of *G. lucidum* (Figure 3).

### 6.1. Precise Gene Editing Technology

Gene editing technology facilitates the modification of specific traits in organisms through precise insertion, deletion, replacement, or base substitution at the genomic level. Methodological advancements in this field have evolved from early zinc-finger nucleases (ZFNs) and transcription activator-like effector nucleases (TALENs) to the currently dominant CRISPR/Cas9 system [120]. In *G. lucidum*, the CRISPR/Cas platform provides a powerful solution to long-standing technical bottlenecks, most notably the extremely low efficiency of homologous recombination [121]. The CRISPR/Cas9 system was initially established using the endogenous U6 promoter for sgRNA transcription, along with an HDV ribozyme for accurate sgRNA processing, which together enabled the formation of an efficient in vivo transcription system. Using this platform, targeted knockout of *cyp5150l8* led to an over 90% decrease in the levels of four representative GAs, genetically confirming the essential role of this gene in GTs post-modification. Disruption of *cyp505d13* demonstrated the wide range of the system for functional gene studies in *G. lucidum* [122]. To overcome the limitations of the heterologous expression systems that are unable to reproduce a natural metabolic environment for *G. lucidum*, precise gene editing was conducted in its monokaryotic strains using the CRISPR/Cas9 system based on ribonucleoprotein (RNP). Homologous recombination repair was achieved by introducing donor DNA, followed by single hybridization to generate dikaryotic editing strains with homozygous target loci. Using this approach, *cyp512a3*, a key gene involved in GTs biosynthesis, was successfully identified and functionally validated [20].

The GC content of the *G. lucidum* genome is approximately 55.9%, with a higher concentration of 59.0% within the exon region [33]. The high-GC composition limits the target sequence range of conventional SpCas9, which has specificity towards the NGG protospacer-adjacent motif (PAM) sequence. This limitation restricts editable sites and increases the risk of off-target effects. In comparison, CRISPR/Cas12a (Cpf1) efficiently recognizes a TTTN PAM, giving it wider target range and lower off-target propensity in high-GC genomes. These advantages establish CRISPR/Cas12a as a promising next-generation editing platform for *G. lucidum* [121]. For triterpene metabolic engineering in *G. lucidum*, targeted editing of the *LS* gene with CRISPR-AsCpf1 enhanced 2,3-oxidosqualene accumulation; this effect was achieved by suppressing the competing endogenous GTs biosynthetic pathway, thereby generating a precursor advantage for the biosynthesis of heterologous triterpene sapogenins [80].

Complementing nuclease-based gene editing tools, CRISPR activation (CRISPRa) and CRISPR interference (CRISPRi) systems support reversible regulation of GTs biosynthesis. Prior work in filamentous fungi has demonstrated that the CRISPR/dLbCas12a-VPR-based system can effectively activate silent biosynthetic gene clusters, promoting the identification of new secondary metabolites [123]. By adapting this approach to *G. lucidum*, CRISPRa could be used to turn on the expression of the rate-limiting enzyme genes (*HMGR*, *SQS*), while CRISPRi-mediated repression of competing sterol pathways enables targeted reprogramming of metabolic flow. Further research will also benefit from prime editing, the template-independent genome modification technology whose high precision makes it ideally suited for elucidating gene functions involved in GTs biosynthesis [124]. Together, these technological advancements have greatly improved both the accuracy of gene editing and the range of feasible applications for *G. lucidum* metabolic engineering.

### 6.2. ML and AI-Driven Metabolic Engineering

ML and AI are driving a transition toward predictive, hypothesis-driven engineering of GTs biosynthesis. By systematically analyzing the complex relationship between multiple gene expression levels and product yields, ML enables the rational engineering of metabolic pathways [125]. Mukherjee et al. (2022) [26] developed a promoter-titered combinatorial library of MVA pathway genes in *S. cerevisiae* and employed random forest modeling to quantify the individual contributions of distinct pathway enzymes. The feature importance analysis identified mevalonate kinase (Erg12p) as the major carbon flux bottleneck for the synthesis of the target product: intermediate Erg12p expression maximized product titers, which was explained by feedback inhibition from downstream isoprenoids. In another ML model for predicting carotenoid production, the expression levels of *tHMG1* and *ERG8* were found to be important factors determining the end yield [125]. These data-driven modeling strategies open up a powerful way to address the uncertainties in decoding genotype-phenotype connections. Within the genome of *G. lucidum*, a considerable number of CYP450 genes remain functionally uncharacterized. An AI-assisted ML model built on protein sequences has been proposed to explore CYP450 function in the GTs biosynthesis. High-accuracy structural prediction tools such as AlphaFold2 provide accurate three-dimensional structures of key enzymes, such as CYP450 and OSC [126,127], allowing virtual screening of mutations to change active sites or substrate channels before conducting experiments. Protein language models also provide a theoretical basis for enzyme variant engineering [128]. Wang et al. (2025) [129] employed a combination of computational modeling and experimental approaches to decode the catalytic mechanism of CYP512A13 and rationally redesigned the enzyme for the biosynthesis of different GTs variants.

Recent advancements in ML and spectroscopic technologies have led to the use of computational tools such as genetic algorithms, neural networks, and hyperspectral imaging (HSI) in the non-destructive analysis of *G. lucidum*. HSI combined with ML models (such as PLSR and LSSVM) has been used for the fast, non-destructive quantitation of GTs and polysaccharides in spores of *G. lucidum* [130]. In another application, ATR-FTIR and Raman spectroscopy coupled with ML models have been evaluated for terpenoid quantitation and classification in essential oils from medicinal plants. The FT-IR/SVR technique showed excellent quantitative accuracy (R^2^ = 0.999), while the Raman/SVM model reached a remarkable classification accuracy of 99.7% [131]. All these spectroscopic techniques allow real-time monitoring of the *G. lucidum* fermentation process, which markedly reduces both the cost and time of conventional HPLC analysis.

### 6.3. Organelle Compartmentalization

Eukaryotic cells compartmentalize their complex metabolic pathways inside different subcellular organelles to reduce metabolic crosstalk and increase catalytic efficiency. This concept has been applied in terpene biosynthesis, with peroxisomes, mitochondria, endoplasmic reticulum (ER), and lipid droplets repurposed as terpene-producing micro-factories.

Peroxisomes are particularly suited for heterogeneous pathway compartmentalization, owing to their ability to dynamically modulate both organelle volume and quantity in response to metabolic demand. Dusséaux et al. (2020) [132] directed the MVA pathway to peroxisomes, leading to a 125-fold increase in monoterpene synthesis along with increased effectiveness of CYP450 modification. Expanding on this work, Baker et al. (2025) [133] used an ML approach to identify overexpression targets that increased the functionality of peroxisomes, resulting in a 137% increase in comparison to the wild-type levels. Targeting of the squalene synthesis pathway to peroxisomes led to a 138-fold increase in squalene titers, which could reach up to 11.00 g/L via two-stage fed-batch fermentation. These findings demonstrate that peroxisomes serve dual functions as both terpene synthesis sites and storage compartments [134].

In *S. cerevisiae*, mitochondria harbor abundant acetyl-CoA pools that serve as robust source precursors for the MVA pathway. Therefore, the transfer of the MVA pathway into mitochondria might utilize these natural sources of acetyl-CoA resources and increase terpene production [135]. Yanagibashi et al. (2024) [136] circumvented the issue of the toxicity of pyrophosphate intermediates by balancing enzyme expression. Coupled with the expansion in mitochondrial volume, this dual approach ensured efficient terpenes production. The other native subcellular location for CYP450 enzymes is the ER. Expanding the ER membrane surface area via *INO*_2_ overexpression provides additional binding sites for CYP450, which finally facilitates terpene oxidation [137]. Given that GTs are highly hydrophobic and poorly soluble in the cytoplasm, they often induce feedback inhibition and membrane toxicity. Lipid droplets naturally function as dedicated storage organelles for hydrophobic compounds. Therefore, by localizing the entire pathway from squalene to various GA components to membrane surface of lipid droplets, the reaction distance is reduced through substrate channeling, while achieving in situ storage of triterpenes to mitigate product toxicity [138]. Yu et al. (2020) [139] improved the storage capacity of intracellular lipid droplets by overexpressing *DGA1* in *S. cerevisiae*, which effectively mitigated the cytotoxicity of α-amyrin and ultimately yielded 1107.9 mg/L.

### 6.4. Chassis Reconstruction

Reconstructing the GTs biosynthesis pathway in model microorganisms such as *S. cerevisiae* through heterogeneous chassis reconstruction also offers a promising strategy for efficient GTs production. With the use of synthetic biology approaches, Wang et al. (2025) [27] have mapped the complete biosynthetic pathway of type II GAs and identified enzyme functions, especially CYP512W6 and GlAT. After reconstructing this pathway in *S. cerevisiae*, the engineered strain showed the ability to synthesize more than 30 distinct GAs, with titers being 1–4 orders of magnitude greater than those through normal cultivation processes. Concurrently, the metabolic engineering of the native *G. lucidum* chassis has also been carried out. Through the introduction of plant-derived amyrin-synthases and beta-amyrin 28-monooxygenase, the precursor supply of 2,3-oxidosqualene was successfully increased, while other competing pathways were eliminated using CRISPR-AsCpf1 technology. Hence, gram-scale biosynthesis of oleanolic acid and ursolic acid has been achieved in fermentation tanks, demonstrating the potential of *G. lucidum* as a versatile cell factory for GTs production [80].

Collectively, these technological advances have transformed *G. lucidum* from a cultivation-based medicinal resource into a predictable engineered triterpene-producing cell factory (Table 2). Within this framework, biosynthesis is no longer carried out by passive observation, but instead driven by rational design, predictive modeling, and precise control.

## 7. Concluding Remarks and Future Perspectives

In recent decades, research in GTs biosynthesis has advanced from investigations of isolated biosynthetic steps to the reconstruction of entire metabolic networks and the implementation of system-level regulatory engineering. GTs biosynthesis is precisely controlled by a multi-pathway signal hub and its crosstalk, including ROS, Ca^2+^, NO, and other upstream or downstream signals. The underlying mechanisms involve bidirectional oscillatory loops, positive feedback, and context-dependent cascades that enable the dynamic and precise regulation of GTs production. Functional validation and quantitative characterization of GTs-related transcription factors have provided key targets for metabolic engineering. The application of CRISPR-based editing, heterologous synthesis platforms, and AI-driven technologies has further accelerated the efficient biomanufacturing of GTs.

The current understanding of the core signal pathway largely depends on static, bulk cellular-level measurements. Yet the functional output of a signal molecule is fundamentally determined by its spatiotemporal distribution. The same molecule can produce opposing regulatory effects depending on its subcellular localization (cytoplasm vs. mitochondria) and activity timing during early or late stress phases. Future efforts using genetically encoded fluorescent probes in combination with live-cell imaging can precisely link the spatiotemporal dynamics of signal molecules with their molecular targets. This strategy will advance the understanding from the network-level signal observations to quantifiable and predictive molecular mechanisms.

On the other hand, the application of AI, gene editing, and multi-omics technologies is driving a major revolution in GTs research. Future priority areas include: (i) the establishment of a curated, multi-omics-integrated database for GTs biosynthesis, which aggregates heterogeneous data layers spanning genomics, transcriptomics, proteomics, and metabolomics; (ii) the systematic mapping of genome-wide transcription factor binding repertoires, with a particular focus on unraveling the regulatory mechanisms of post-translational modifications, and the integration of multi-omics with gene editing to build a holistic model linking signal perception to GTs production; and (iii) the development of deep learning models to predict enzyme functions, facilitating enzyme engineering and optimize product yields. Overall, these advancements will allow this valuable medicinal fungus to move beyond its traditional role. This paradigm shift will provide new opportunities and robust technical platforms for the sustainable production of GTs.

## Figures and Tables

**Table 1:** Summary of transcription factors involved in the regulation of GTs biosynthesis in *G. lucidum*.

Transcription Factors	Family	Regulatory Effects	Primary Regulation Mechanisms on GTs Biosynthesis	References
SREBP	bHLH-zip	Positive (13 different GAs increased significantly)	*SREBP* binds to the promoter regions of target genes (*HMGS*, *MVK*), and enhances their transcription levels.	[23]
*Gl*bHLH5	bHLH	Positive (up approximately 21–45% of GTs)	*GlbHLH5* binds to the P2 region of the *LS* promoter and activates its expression.	[99,101]
*Gl*bHLH7	bHLH	Positive	*GlbHLH7* binds to the *SE* and *SQS* promoters and activates their expression.	[99]
Zn2Cys6_61	Zn_2_Cys_6_	Positive (up approximately 16.9–32.4% of GTs)	*Zn*_2_*Cys*_6_*_61* binds to and activates the promoter of *SQS*.	[28]
Zn_2_Cys_6__82	Zn_2_Cys_6_	Positive	Zn_2_Cys_6__82 upregulates the expression of key genes (*HMGR*, *SQS*, and *LS*), with *SQS* showing the greatest transcriptional induction.	[91]
HMG_9	HMG-box	Positive	*GlHMG_9*-silencing significantly reduces the expression of *GlHMG_9* and *LS*, accompanied by the downregulation of several genes associated with GTs biosynthesis, such as *MPK* and *CYP5144F11*.	[82]
C_2_H_2_.07	C_2_H_2_ zinc finger	Positive	*C*_2_*H*_2_*.07* overexpression increases the expression levels of *LS*, *HMGR* and *SQS*. While *C2H2.07-*silencing significantly reduces the expression of several genes involved in GTs biosynthesis, such as *MPK*, *FPS*, *LS*, *Zn*_2_*Cys*_6_*_37*, *CYP5035M2*, *CYP5144C41*, *CYP5144C54*, and *CYP5147B1*.	[82]
*Gl*CRZ1	C_2_H_2_ zinc finger (calcineurin-responsive)	Positive (for Ca^2+^ signal transduction)	glcrz1 is essential for Ca^2+^ signal transmission by enabling cells to perceive Ca^2+^ stimulation.	[84]
*Gl*CRZ2	C_2_H_2_ zinc finger (calcineurin-responsive)	Positive (for basal synthesis) (Δ*glcrz2* decreasing individual GTs to 0.012–0.20 mg/g, much lower than WT (0.56–3.3 mg/g)	Knockout of *glcrz2* severely impairs mycelial growth and GTs accumulation.	[84]
PacC	C_2_H_2_ zinc finger (pH-responsive)	Negative	*GlPacC*-silencing significantly increases the transcript levels of *SQS* and *LS*, accompanied by abnormal mycelial growth.	[86]
*Gl*Swi6	APSES	Positive (*GlSwi6-*silencing induces 25% reduction in GTs)	*Gl*Swi6 regulates GTs biosynthesis by modulating the intracellular ROS levels, affecting both the activity of ROS-related enzymes and the expression of their corresponding genes.	[22]
*Gl*Myb	MYB	Positive (Overexpression of *GlMyb* increases 29.1–35.4% of GTs)	*GlMyb* induces the expression of *spds1* and *spds2* by directly binding to their promoters, while *Spd* is crucial for the GTs accumulation under heat stress.	[112]
LaeA	Methyltransferase	Positive (Overexpression of *LaeA* increases GA-T (1.25-fold) and GA-Me (1.20-fold))	Δ*laeA* drastically reduces the transcripts of *sqs* and *ls.*	[74]
*Gl*Slt2	-	Positive (*GlSlt2*-silencing induces 35–40% reduction in GTs)	*Gl*Slt2 regulates GTs biosynthesis via modulating the intracellular ROS level, including ROS-related genes and enzymes (SOD, CAT).	[113]
*Gl*Tert	Telomerase reverse transcriptase (non-canonical)	Positive (*GlTert*-silencing induces 46–49% reduction in GTs)	*Gl*Tert regulates the intracellular ROS balance by altering the antioxidant status, subsequently modulating the expression of key genes (*hmgr*, *sqs*, *osc*).	[114]
*Gl*Sirt1	Sirtuin (NAD^+^-dependent deacetylase)	Positive (*GlSirt1*-silencing induces 32.6–41.8% reduction in GTs)	Exhibits *Gl*sirt1-mediated deacetylation of *Gl*CAT.	[43,115]
GCN4	bZIP	Negative (under N-limitation) *(GCN4*-silencing induces 1.51–2.2 folds increase in GTs)	*GCN4* binds to and activates the promoters of antioxidant enzyme genes (*gr*, *gst2*, *and cat3*), reducing ROS levels and suppressing GAs overaccumulation.	[105]
AreA	GATA	Dual (positive/negative)	*AreA* binds to and activates the promoter of *fps*, and it also regulates the expression of *NR* to influence intracellular NO content.	[21]
*Gl*MADS1	MADS-box	Negative	*GlMADS1-*silencing upregulates the transcription levels of *hmgs*, *hmgr*, *fps*, *sqs*, with most significant change occuring in *fps*.	[109]
Skn7	response regulator (HSF-like)	Negative	*Gl*Skn7 regulates the ROS levels and antioxidant enzymes, which subsequently affects the gene expression of *hmgr*, *osc*, *sqs*.	[116]
*Gl*HSF2 (HSF family)	HSF	Differential (likely positive for downstream)	Hypothesized differential regulation: *Gl*HSF2 inhibits the expression of upstream rate-limiting genes *HMGR* and *HMGS* while activating downstream genes *IDI*, *PMK*, and *MVD*, thereby supporting the supply of downstream precursors (e.g., IPP and DMAPP) for GTs biosynthesis.	[117]
14-3-3 proteins (*Gl*Bmh1/*Gl*Bmh2)	14-3-3 (phosphopeptide-binding)	Negative	Double silencing upregulates the transcription levels of *sqs* and *osc*.	[118]
PRMT5	Arginine methyltransferase (epigenetic)	Negative (*PRMT5*-silencing induces 1.4–1.48 folds increase in GTs)	*Gl*PRMT5 weakens *Gl*PP2C1 activity by dimethylating its arginine 99 (R99) and arginine 493 (R493) residues, whereas *GlPP2C1-*silencing downregulates the gene expression of *sqs*.	[119]
Homeobox TF (GL25472)	Homeobox	Positive (Its silencing induces 28.9–42.3% reduction in GTs)	*GL25472* binds the promoter regions of *hmgr* and *osc*, and its silencing downregulates the expressions of *hmgr*, *sqs*, and *osc*.	[40]
Velvet proteins (VeA, VelB, VelC)	Velvet	Positive	Silencing of *VeA* or *VelB* downregulates the expression of *hmgr*, *sqs*, and *osc*.	[40]

**Table 2:** Comparative assessment of engineering strategies for enhancing GTs biosynthesis.

Strategy	Technical Feasibility	Expected Improvement in GTs Yield	Implementation Complexity	Cost–Benefit Considerations	Scalability	Current Stage of Development
Precise gene editing	CRISPR/Cas9 has been established in *G. lucidum*, and Cas12a (AsCpf1) has been applied to edit the *LS* gene, so gene editing is technically straightforward in this fungus. One remaining constraint is that precise editing of endogenous loci still depends on monokaryotic strain manipulation followed by hybridization.	Gene editing has limited potential for increasing GTs yields; its benefits are largely qualitative, serving mainly for functional validation and metabolic-flux redirection rather than for large-scale increases in titer.	Single-gene editing has been demonstrated for multiple CYP450 targets.	It is favorable in the long term, since the edited strains are stably heritable with no recurring costs.	The edited trait can be maintained through clonal propagation and fermentation scale-up.	This approach is well established in *G. lucidum*; multi-gene functional validation has already been achieved, and the technology is ready for deployment.
ML/AI-guided metabolic engineering	This approach is constrained by uncharacterized CYP450 and scarce training data, although structure prediction tools such as AlphaFold2 partly offset this limitation.	It contributes to GTs yields mainly by locating flux bottlenecks and guiding rational enzyme design rather than directly raising yield.	It requires computational modeling coupled with iterative wet-lab validation.	The upfront modeling cost can be partly offset by reduced trial-and-error and lower analytical burden.	The models generalize poorly across targets and usually require retraining per application.	This strategy is still at the early proof-of-concept stage in *G. lucidum*; the demonstrations reported so far have come largely from *S. cerevisiae*.
Organelle compartmentalization	This strategy is well established in *S. cerevisiae* but has not yet been established in *G. lucidum*; it requires coordinated engineering of signal peptides, metabolite transport, and storage capacity.	It has achieved order-of-magnitude titer gains in the yeast host.	It demands multi-part, coordinated pathway engineering and flux balancing.	The economic return is currently restricted to yeast.	Yeast strains have already reached bioreactor scale, whereas the corresponding capability in *G. lucidum* remains unvalidated.	This strategy has matured considerably in yeast but remains essentially unexplored in *G. lucidum*, representing a proven toolkit awaiting transfer.
Chassis reconstruction	The heterologous route is technically straightforward because *S. cerevisiae* is a mature and well-characterized chassis, whereas the native *G. lucidum* chassis is constrained by its low homologous-recombination efficiency.	Order-of-magnitude gains in GTs productions have been achieved in the heterologous (yeast) chassis, whereas the native *G. lucidum* chassis has reached gram-scale production of the triterpenoids oleanolic and ursolic acid.	It requires complete pathway resolution and flux balancing.	It offers considerable cost–benefit potential, since short fermentation cycles lower downstream costs.	*S. cerevisiae* is already an industrialized host, and the native chassis has reached gram-scale fermenter production.	This approach has advanced rapidly and has already been demonstrated at the gram scale.

## Data Availability

No new data were created or analyzed in this study. Data sharing is not applicable to this article.

## Abbreviations

The following abbreviations are used in this manuscript:

GTs	Ganoderma triterpenoids	MVA	mevalonate
ROS	reactive oxygen species	Ca^2+^	calcium ions
NO	nitric oxide	CRISPR/Cas9	clustered regularly interspaced short palindromic repeats/CRISPR-associated protein 9
RNAi	RNA interference	DAP-seq	DNA affinity purification sequencing
AI	artificial intelligence	ML	machine learning
AACT	acetyl-CoA acetyltransferase	HMGR	3- hydroxy-3-methylglutaryl-CoA reductase
HMGS	3-hydroxy-3-methylglutaryl-CoA synthase	FPS	farnesyl-diphosphate synthase
SQS	squalene synthase	LS	lanosterol synthase
CYP450	cytochrome P450 monooxygenases	DEGs	differentially expressed genes
HADH	3-hydroxyacyl-CoA dehydrogenase	DXS	1-deoxy-D-xylulose-5-phosphate synthase
PMVK	phosphomevalonate kinase	ACAT	acetyl-CoA acetyltransferase
*SQLE*	squalene epoxidase	TCA	tricarboxylic acid cycle
WGCNA	weighted gene co-expression network analysis	ETC	mitochondrial electron transport chain
Nox	NADPH oxidases	MeJA	methyl jasmonate
SA	salicylic acid	AOX	alternative oxidase
GPx	glutathione peroxidase	GSNOR	S-nitrosoglutathione reductase
GA	ganoderic acid	CAT	catalase
H_2_S	hydrogen sulfide	CAM	calmodulin
cna	calcineurin subunit A	cch	calcium-permeable channel gene
plc	hosphoinositide-specific phospholipase	ABA	abscisic acid
NOS	nitric oxide synthase	NR	nitrate reductase
c-PTIO	2-(4-carboxyphenyl)-4,4,5,5-tetramethylimidazoline-1-oxyl 3-oxide	SNP	sodium nitroprusside
cGMP	cyclic guanosine monophosphate	NAC	N-acetylcysteine
SOD	superoxide dismutase	APX	ascorbate peroxidase
GSH	glutathione	MDA	malondialdehyde
EGTA	ethylene glycol-bis(2-aminoethyl ether)-N,N,N′,N′-tetraacetic acid	LaeA	loss of *aflR* expression-A
CRZ1	calcineurin-responsive transcription factor 1	CRDEs	calcineurin-dependent response elements
bHLH	basci helix-loop-helix	SREBP	sterol regulatory element-binding protein
mTOR	mammalian target of rapamycin	GCN4	general control nonderepressible 4
PAM	protospacer-adjacent motif	HSI	hyperspectral imaging
PLSR	partial least squares modeling	LSSVM	least squares support vector machine
ATR-FTIR	Aattenuated total reflectance—fourier transform infrared spectroscopy	ER	endoplasmic reticulum
